# Neurophysiological and psychophysical effects of dry versus sham needling of the infraspinatus muscle in patients with chronic shoulder pain: a randomized feasibility study

**DOI:** 10.1186/s40945-021-00118-x

**Published:** 2021-10-18

**Authors:** Antoine Laramée, Guillaume Léonard, Mélanie Morin, Mélanie Roch, Nathaly Gaudreault

**Affiliations:** 1 University of Sherbrooke, School of Medicine and Health Sciences, School of Rehabilitation, Centre de Recherche du Centre Hospitalier Universitaire de Sherbrooke (CRCHUS), 3001, 12e Avenue Nord, Sherbrooke, Québec, Canada; 2 University of Sherbrooke, School of Medicine and Health Sciences, School of Rehabilitation, Centre de Recherche sur le Vieillissement (CdRV), 1036 Rue Belvédère S, Sherbrooke, Québec, Canada

**Keywords:** Dry needling, Sham needling, Myofascial trigger point, Neurophysiological effect, Transcranial magnetic stimulation, Pressure pain threshold

## Abstract

**Background:**

Dry needling (DN) is increasingly used for treating myofascial trigger points (MTrPs) and has shown significant effects on pain and function. This study aimed to assess feasibility of conducting a randomized sham-controlled trial and to collect preliminary data on the effects of infraspinatus DN on corticospinal excitability and mechanical pain sensitivity.

**Method:**

This randomized feasibility study included adults with chronic non-traumatic shoulder pain and a infraspinatus MTrP. Participants were randomized to receive real DN or sham DN in the infraspinatus MTrP. Feasibility outcomes included data pertaining to recruitment, retention of participants, completeness and safety of assessment procedures. Neurophysiological and psychophysical outcomes included corticospinal excitability and mechanical pain sensitivity measured by active motor threshold (aMT) and pressure pain threshold (PPT), respectively. They were assessed at baseline, immediately after and 24 h post-intervention.

**Results:**

Twenty-one participants were recruited over a 6-month period. Nineteen participants completed the treatment and follow-up assessment. Motor evoked potential responses were discernible in all but 1 participant. Only 1 minor adverse event related to transcranial magnetic stimulation (mild headache) affected the measurements. No DN adverse effects were recorded in both groups. An overall completeness rate of 81% was reached, with 70% completeness in the DN group and 91% in the sham group. Data analysis revealed that real DN increased corticospinal excitability (reduced aMT) 24 h post-intervention (Mdn = − 5.96% MSO, IQR = 5.17, *p* = 0.04) and that sham DN triggered similar responses immediately after the intervention (Mdn = − 1.93% MSO, IQR = 1.11, *p* = 0.03). Increased mechanical pain sensitivity (reduced PPT) was significant only in the sham group, both immediately (Mdn = − 0.44 kg/cm^2^, IQR = 0.49, *p* = 0.01) and 24 h post-intervention (Mdn = − 0.52 kg/cm^2^, IQR = 1.02, *p* = 0.02). Changes in corticospinal excitability was positively correlated with changes in mechanical pain sensitivity in the DN group, both immediately (r = 0.77, p = 0.02) and 24 h post-intervention (r = 0.75, *p* = 0.05).

**Conclusion:**

The present study demonstrates the feasibility of quantifying the neurophysiological and psychophysical effects of DN, and provides recommendations and guidelines for future studies. Moreover, it provides preliminary evidence that DN may increase corticospinal excitability of the infraspinatus muscle in patients with chronic shoulder pain and that the relationship of neurophysiological and psychophysical effects is promising to better understand its mechanisms of action.

**Trial registration:**

NCT04316793; retrospectively registered November 3, 2020.

## Introduction

Myofascial pain syndrome is a common musculoskeletal disorder associated with the presence of myofascial trigger points (MTrPs) [1, 2]. MTrPs are defined as hypersensitive spots associated with a palpable nodule in a taut skeletal muscle band that are painful on compression and which can evoke referred pain [3–5]. It has been suggested that MTrPs are the source of pain for a large proportion of patients consulting in primary care clinical settings [1–3]. The prevalence of muscles containing MTrPs is very high in people suffering from chronic shoulder pain [6–8] since up to 77% of this population have MTrPs in the infraspinatus [6]. These shoulder MTrPs have been associated with pain in the neck, upper back and shoulder region [6–8] as well as with activity limitations [6].

Dry needling (DN) is increasingly used for treating MTrPs [9–11]. It consists in the insertion of fine monofilament needles, similar to those used in acupuncture, within the MTrP [2, 9–11]. Evidence of the clinical effects has been reported after DN, mainly in the short term [2, 10, 12–15], and includes decreased pain [2, 10, 12–17], increased range of motion [12, 16] and increased function, [10, 13, 14, 16, 18]. The underlying mechanisms of action are still unclear and the possibility that these clinical effects are related to placebo cannot be ruled out [19]. Most of our knowledge of the neurophysiologic effects of DN comes from the literature in traditional acupuncture. However, DN applied to MTrPs differs from traditional acupuncture [20]. Contrary to traditional acupuncture, DN is not based on a conceptual framework involving meridians with predetermined points for needle insertion [10, 20]. Thus, the hypotheses derived from traditional acupuncture literature about the neurophysiological effects can hardly be extended to DN, at least from a conceptual perspective. To date, very few studies have been conducted to specifically investigate DN [20, 21] and the few observations that shed light on its neurophysiological effects mainly comes from animal studies [22], and the effect of DN on psychophysical measures such as mechanical pain sensitivity has yet to be demonstrated in humans with valid, accurate and sensitive tools [10, 20].

Recent evidence suggests the clinical benefits of DN could be related to neurophysiological changes involving the central nervous system (CNS) [20, 23]. Transcranial magnetic stimulation (TMS) is a safe and non-invasive technique that can be used to evaluate corticospinal pathways and other CNS functions [24, 25]. A number of studies have used TMS to outline the effect of experimental and clinical pain on the motor cortex (M1) and the associated corticospinal pathways. One highly relevant example of this comes from the study of Ngomo et al. (2015) [26] using TMS to investigate corticospinal excitability of the rotator cuff muscles in patients with unilateral rotator cuff tendinopathy. Results showed that these patients exhibited a decrease in corticospinal excitability in the hemisphere corresponding to the affected limb, suggesting that corticospinal excitability changes may be a significant pathophysiological hallmark of rotator cuff tendinopathy. To the best of our knowledge, no studies have examined the effect of DN on corticospinal excitability. Therefore, the feasibility of a study measuring the effects of DN on corticospinal excitability remains to be demonstrated.

The aims of this study were: (1) to assess the feasibility of a protocol measuring infraspinatus corticospinal excitability following a DN intervention; (2) to collect preliminary data on neurophysiological (corticospinal excitability) and psychophysical (mechanical pain sensitivity) effects of DN immediately and 24 h after treatment in an infraspinatus MTrP as compared with sham DN; and (3) to explore the relationship between DN-induced changes in neurophysiological and psychophysical outcomes.

## Methods

### Design

In this double-blind feasibility study (Clinical Trial Registry Identifier: NCT04316793), 21 adults with chronic non-traumatic shoulder pain were randomized into two groups: one group receiving real DN (*n* = 10) and another group receiving sham DN (*n* = 11). Corresponding sample size (10 participants per group) is acceptable and recommended for this type of study [27, 28]. To compensate potential losses to follow-up, an additional participant has been recruited during the study (randomized in the sham DN group). Participants and evaluators were blinded to group allocation. Real and sham DN were applied as described below. Measurements were taken at baseline (T1), immediately after the intervention (T2), and 24 h after the intervention (T3). The study protocol was approved by the Ethics Committee of the CIUSSS de l’Estrie – CHUS (Registration No. 2019–3133).

### Participants

Participants had to meet the following inclusion criteria: (1) unilateral, chronic non-traumatic shoulder pain (VAS ≥ 1/10; > 3 months); (2) localized pain in the shoulder region or referred pain according to the territory of the infraspinatus [5]; (3) presence of a palpable nodule inside a taut muscle band reproducing the patient’s pain. To ensure that DN was safe for participants, we excluded individuals with osteoporosis or excessive atrophy of the infraspinous fossa (infraspinatus < 10 mm), and cancer or metastasis in organs or tissues above the pelvis (< 5 years). As for TMS procedure safety, we excluded individuals with neurological, psychiatric or epilepsy conditions; presence of metal or electronic implants, or a metallic foreign body in the eye; history of head trauma with loss of consciousness; and pregnant women. Individuals with the following confounding diseases or conditions were also excluded: shoulder capsulitis; shoulder, thorax or mastectomy surgeries; shoulder bone fracture (< 6 months); C4-C5 or C6 radiculopathy. Lastly, we excluded individuals who had previously received DN treatment to ensure that the participants would remain blinded to the intervention.

### Procedures

Participants were recruited via advertisements placed on bulletin boards in the Faculty of Medicine and Health Sciences at the Université de Sherbrooke and in physiotherapy clinics in the Eastern Townships region. Recruitment took place from August 2019 to December 2019. Interested individuals were invited to contact the research assistant in charge of the study to verify the eligibility criteria (see below). Those meeting the criteria were then invited to an initial appointment in our laboratory, located at the Research Center on Aging in Sherbrooke (Quebec, Canada). Upon arrival, participants were greeted by a research assistant who explained the nature of the project, obtained written informed consent and verified the remaining eligibility criteria. The presence of MTrPs in the infraspinatus was confirmed by a physiotherapist with more than 20 years of experience in the identification of MTrPs, according to the following standardized procedure [29]: the individual was asked to lie in a side-lying position on a treatment table on the asymptomatic side. The upper arm was supported by a pillow placed in front, so that the shoulder muscles were relaxed; the arm was positioned in slight horizontal adduction to slightly stretch the fibers of the infraspinatus muscle. Manual palpation perpendicular to the infraspinatus muscle fibers was used to identify the tight muscle band. Once a taut muscle band was identified, the physiotherapist searched within this band for a contraction node, namely the MTrP. The physiotherapist then validated with the patient if the compression of the MTrP reproduced local or referred pain. This pain had to correspond to the pain patterns known to occur with the infraspinatus MTrP according to Simons et al. (1999) [5] and to reproduce the participant’s pain symptoms. The pain intensity should be at least 1/10 on a visual analog scale (VAS) where 0/10 = *no pain* and 10/10 = *worst pain imaginable*. The evaluator then identified the location of this MTrP with a non-toxic black Sharpie pen. Individuals for whom no MTrPs were identified received advice from the physiotherapist about sleep positions and movements to avoid or modify during daily activities. They were also advised to consult a healthcare professional according to their condition. When relevant, instructions were given on how to find a physiotherapist in their region. Individuals who met this final inclusion criteria (presence of MTrP in the infraspinatus muscle) completed the questionnaire used to confirm that no DN or TMS contraindication was present and to collect baseline medical information. Participants were randomly assigned to one of the two intervention groups using a random number generator (MS Excel software) run by the principal investigator and went through the baseline assessment (T1).

Both real and sham DN interventions and procedures were performed according to current recommendations issued by the regulating authorities governing physiotherapy practice in Quebec, the OPPQ. The physiotherapists involved in treatments were experienced in DN and certified by the OPPQ, which involves over 102 h of training in DN [30]. Prior to the intervention, the physiotherapist explained the purpose of DN and reviewed the associated contraindications and precautions. She then inserted a sterile disposable acupuncture needle (OPTIMED, non-silicone, 40 mm × 0.30 caliber) in the MTrP. The direction of the needle was slightly oblique and in the direction of the muscle fibers. If necessary, a pistoning technique was used to try and elicit a local twitch response (LTR) [31]. The needle was then immediately removed. The same needle position and direction was used for the sham group. The needle was inserted at the subcutaneous level, at the depth of the superficial adipose tissue. The needle was held there for a couple seconds without any manipulation and was then removed. During the intervention, participants in the DN group and the sham group were placed in the same position as for the MTrP evaluation described above.

### Feasibility outcomes

Throughout the study, descriptive data were collected to assess the following feasibility outcomes: (1) exclusion rate and exclusion criteria associated with each excluded individual (e.g. osteoporosis); (2) refusal rate and reason for refusal; (3) recruitment rate; (4) retention rate and if possible, the reason for loss at follow-up; (5) duration of the procedure; (6) completeness: participants for whom data on corticospinal excitability and mechanical pain sensitivity for T1,T2 and T3 were collected; (7) adverse effects rate and safety of the procedure: frequency, type and severity of any adverse effects.

### Neurophysiological outcomes

Corticospinal excitability was assessed with transcranial magnetic stimulation (TMS). TMS is a reliable and safe method to assess the excitability and integrity of M1 and the corticospinal tract [26, 32–35]. In this study, active motor threshold (aMT), expressed in maximum stimulator output percentage (%MSO), was measured using a Magstim 200 TMS stimulator (Magstim Company Ltd., United Kingdom) connected to a 70-mm figure-of-eight coil. A Brainsight neuronavigation device (Rogue Research, Montreal, Canada) was used to ensure precise positioning of the coil over the head of each participant. Stimulation target location was fine-tuned for each participant to stimulate the M1 hotspot, defined as the optimal site for eliciting motor evoked potentials (MEPs) in the contralateral infraspinatus with the lowest stimulation intensity.

MEPs were recorded from electromyographic (EMG) recording of the infraspinatus, with surface electrodes placed 3 cm below and running parallel to the scapula spine, over the infraspinatus fossa. The aMT was defined as the minimal TMS intensity required to produce discernible MEP amplitudes from the background EMG in at least 50% of the trials [26, 36]. During this procedure, participants had to perform an isometric external rotation movement and hold the muscle contraction at 7.5% ± 2.5% of their isometric maximal voluntary contraction (maximal value of two trials measured previously with a dynamometer; 30 s rest period between the two trials).

### Psychophysical outcomes

Mechanical pain sensitivity was assessed with a pressure algometer. Pressure algometry is a reliable method for assessing pressure pain threshold (PPT), a parameter used to measure a MTrP treatment’s effect [37]. In this study, PPT, expressed in kg/cm^2^, was defined as the mean value of three measurements taken at 30-s intervals and was measured using a Force Ten™ FDX (Wagner instrument, Greenwich, USA) equipped with a 1 cm^2^ probe and directly applied on the MTrP. Once again, participants were placed in the same position as for the MTrP evaluation.

### Statistical analysis

For baseline demographics, clinical characteristics, and feasibility outcomes, descriptive statistics (such as the mean ± standard deviation or median [interquartile range] for continuous variables and frequency percentage for categorical variables) were used. To examine the effects of DN, analysis with Wilcoxon signed-rank tests were used to assess within-group changes, and Mann-Whitney tests were used to identify between-group differences. The magnitude of aMT and PPT (delta scores) were measured by subtracting the average baseline (T1) score from the average post-intervention (T2; T3) score, such that a negative value indicated an increase in corticospinal excitability (reduced aMT) or an increase in mechanical pain sensitivity (reduced PPT). We used r-value = Z/√N to calculate the sham and DN intra-group effect size at each time point for corticospinal excitability and mechanical pain sensitivity [38]. Lastly, Spearman’s rank correlation coefficients were used to assess the relationships between corticospinal excitability of the infraspinatus and PPT, and also between delta scores (between T1 and T2, and between T1 and T3) for these same variables. All analyses were performed with IBM SPSS 26.0 and the level of significance was set at α = 0.05. Missing data (including lost to follow-up) were withdrawn from the analyses (listwise deletion).

## Results

Participant demographics and baseline clinical characteristics are shown in Table 1. No statistical differences were found between the two groups.

**Table 1:** Baseline demographics and clinical characteristics of study participants

Characteristics	DN group (*n* = 10)	Sham DN group (*n* = 11)	Total (*n* = 21)	***p***-value
**Age (years)**	**n (%)**	**n (%)**	**n (%)**	0.072
Mean ± SD	36.6 ± 14.8	47.2 ± 13.2	42.1 ± 14.6	
**Gender**	**n (%)**	**n (%)**	**n (%)**	0.557
Male	8 (80)	7 (64)	15 (71)	
Female	2 (20)	4 (36)	6 (29)	
**Dominant hand**	**n (%)**	**n (%)**	**n (%)**	0.973
Right	9 (90)	10 (91)	19 (90)	
Left	1 (10)	1 (9)	2 (10)	
**Painful shoulder**	**n (%)**	**n (%)**	**n (%)**	0.654
Right	7 (70)	9 (82)	16 (76)	
Left	3 (30)	2 (18)	5 (24)	
**Pain onset (months)**	**n (%)**	**n (%)**	**n (%)**	0.705
3–6	2 (20)	2 (19)	4 (19)	
6–12	3 (30)	5 (45)	8 (38)	
12–24	1 (10)	1 (9)	2 (10)	
> 24	4 (40)	3 (27)	7 (33)	
**Smoking history**	**n (%)**	**n (%)**	**n (%)**	0.180
Never	10 (100)	7 (64)	17 (81)	
Quit smoking	0 (0)	4 (36)	4 (19)	
Currently smoking	0 (0)	0 (0)	0 (0)	
**Physical activity** (sessions per week)	**n (%)**	**n (%)**	**n (%)**	0.679
< 1	1 (10)	4 (36)	5 (24)	
1–3	5 (50)	2 (19)	7 (33)	
> 3	4 (40)	5 (45)	9 (43)	
**Visual analog score** (/10) (at rest)				
Mean ± SD	1.1 ± 1.6	1.3 ± 2.4	1.2 ± 2.0	0.973
Range	0–4	0–8	0–8	
**Visual analog score** (/10) (during activities/movement)				
Mean ± SD	6.4 ± 1.3	6.0 ± 2.2	6.2 ± 1.8	0.973
Range	4–8	1–8	1–8	

### Feasibility outcomes

**Fig. 1:**
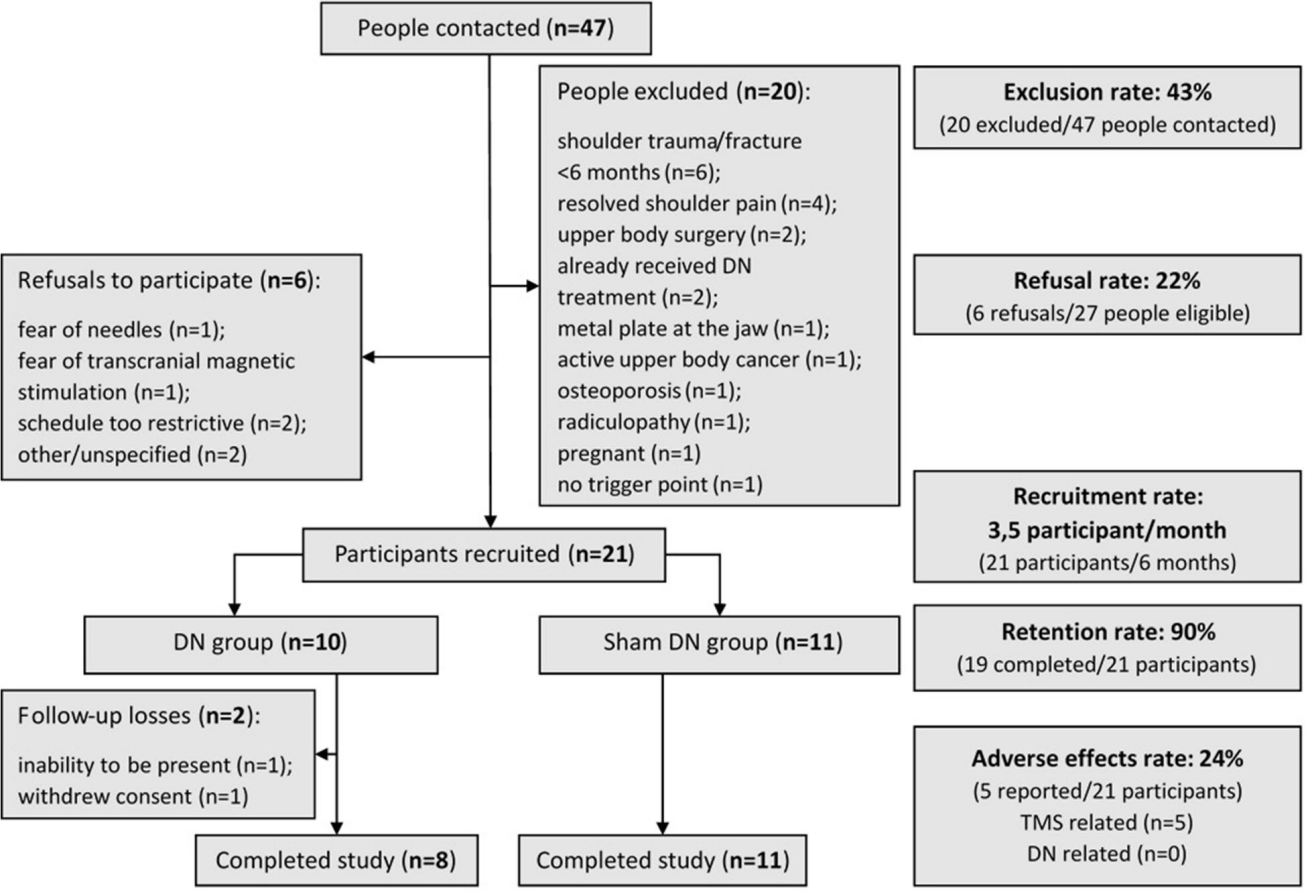
Flow diagram showing enrollment, group randomization and follow-up DN: Dry needling; TMS: Transcranial magnetic stimulation

Over a period of 6 months, forty-seven individuals showed interest and were contacted by the research assistant (Fig. 1).

(1) Exclusion rate: Twenty of these forty-seven individuals (43%) were excluded from the study (see Fig. 1). Thirteen were excluded due to a concurrent medical condition, two were excluded because they had already received dry needling treatments for their condition and another four were excluded due to resolved shoulder pain before the baseline assessment. One individual was excluded at the initial appointment because we were unable to identify an infraspinatus MTrP.

(2) Refusal rate: Of the 27 eligible individuals, six (22%) refused to participate. Reasons for refusal included fear of needles (*n* = 1), apprehension regarding TMS (*n* = 2) and time constraints that did not allow the individuals to attend two sessions within a 24-h period (n = 2). Two individuals declined without specifying a reason.

(3) The recruitment rate averaged 3.5 participants per month; 21 participants were recruited in a 6-month period and were randomized into two groups: the experimental group (real DN, *n* = 10) and the control group (sham DN, *n* = 11).

(4) Retention rate and reason for loss at follow-up: Two losses at follow-up were recorded in the experimental group: 1 participant could not attend the 24-h post-intervention evaluation due to a snowstorm and 1 participant withdrew from the study after experiencing a mild headache which occurred after T2.

(5) Duration of the procedure (mean ± SD): 42.6 min ± 14.5 per measurement time.

(6) Completeness: Considering losses to follow-up, 8 participants in the DN group and 11 participants in the sham DN group completed PPT measurements (T1-T2-T3). In the DN group, we were unable to produce discernible MEP amplitudes with TMS in 1 participant. In the sham DN group, 1 participant remembered having a contraindication to TMS, which he forgot to specify in the screening questionnaire. Therefore, of these 8 participants in the DN group and 11 participants in the sham DN group, 7 and 10 participants completed the TMS assessment, respectively.

(7) Safety of the procedure and adverse effects: A total of 5 adverse effects were reported: 1 participant experienced a mild headache (same participant recorded as a loss at follow-up; previously experienced pain pattern), 4 participants reported difficulties maintaining the position when completing the TMS assessment (increased shoulder pain due to sustained shoulder external rotation); nevertheless, these 4 participants were all able to complete the assessment, and only 1 of them reported moderate discomfort that persisted 24 h after the evaluation. No adverse effects due to DN were recorded.

### Neurophysiological outcome

#### Corticospinal excitability

In the real DN group, within-group analyses revealed that the change in corticospinal excitability (increased excitability) observed from T1 to T2 did not reach statistical significance (*p* = 0.08) whereas a significant increase in corticospinal excitability (as demonstrated by reduced aMT) was observed between T1 and T3 (*p* = 0.04). With regard to the sham group, within-group analyses revealed a significant increase in corticospinal excitability (reduced aMT) between T1 and T2 (*p* = 0.03) whereas no changes were observed from T1 to T3 (*p* = 0.19). Between-group analyses (Mann-Whitney U tests), comparing changes between the DN and sham group, revealed no significant differences between T1 and T2 (*p* = 0.52) or between T1 and T3 (*p* = 0.16; see Table 2).

**Table 2:** Neurophysiological and psychophysical outcomes

	Corticospinal excitability – Transcranial magnetic stimulation									
	Within group								Between group	
	DN group **T**_**1**_ **Mdn [IQR] =** 49.90 [7.31] ^a^				Sham DN group **T**_**1**_ **Mdn [IQR] =** 50.17 [7.55] ^a^					
	**Z**	**p-value**	**Mdn [IQR]**	**ES**	**Z**	**p-value**	**Mdn [IQR]**	**ES**	**U**	**p-value**
**T**_**1**_**-T**_**2**_	−1.481	0.08	−2.61 [4.95] ^a^	−0.49	−1.886	0.03*	−1.93 [1.11] ^a^	− 0.60	45.0	0.52
**T**_**1**_**-T**_**3**_	−1.859	0.04*	−5.96 [5.17] ^a^	−0.70	−0.968	0.19	−0.52 [2.07] ^a^	− 0.31	24.0	0.16
	Pressure pain – Algometer									
	Within group								Between group	
	DN group **T**_**1**_ Mdn [IQR] **=** 7.19 [3.39] ^b^				Sham DN group **T**_**1**_ Mdn [IQR] **=** 8.47 [2.64] ^b^					
	**Z**	**p-value**	**Mdn [IQR]**	**ES**	**Z**	**p-value**	**Mdn [IQR]**	**ES**	**U**	**p-value**
**T**_**1**_**-T**_**2**_	−0.051	0.50	−0.18 [0.31] ^b^	−0.02	−2.223	0.01*	−0.44 [0.49] ^b^	− 0.67	25.5	0.02*
**T**_**1**_**-T**_**3**_	−1.260	0.13	−0.62 [0.87] ^b^	−0.45	−2.134	0.02*	−0.52 [1.02] ^b^	− 0.64	34.0	0.22

^a^Results are expressed as a percentage of the maximum stimulator output (%MSO); ^b^Results are expressed in kg/cm2; *Statistically significant; T1: Baseline; T2: Immediately post-intervention; T3: 24 h post-intervention; Mdn: Median; IQR: Interquartile range; ES: Effect size expressed by r-value = Z/√N

### Psychophysical outcome

#### Mechanical pain sensitivity at the trigger point

Within-group analyses revealed that participants assigned to the real DN group showed no significant difference between T1 and T2 (*p* = 0.50) or between T1 and T3 (*p* = 0.13). Conversely, participants in the sham group showed a significant increase in mechanical pain sensitivity (as demonstrated by reduced PPT) between T1 and T2 (*p* = 0.01) and between T1 and T3 (*p* = 0.02). Between-group analyses (Mann-Whitney U tests) revealed that these mechanical pain sensitivity differences between the DN and sham group were significant between T1 and T2 (p = 0.02) but were not significant between T1 and T3 (*p* = 0.22; see Table 2).

### DN and sham effect size

#### Corticospinal excitability

Both DN and sham DN showed a large effect size (ES) on corticospinal excitability immediately after the intervention (T1-T2; r = − 0.49 and − 0.60 respectively). DN effect size increased 24 h after intervention (T1-T3; r = − 0.70) while sham effect size decreased to a medium effect size (r = − 0.31).

#### Mechanical pain sensitivity at the trigger point

DN showed a very small effect size to increase mechanical pain sensitivity immediately after the intervention (T1-T2; r = − 0.02) but nearly had a large effect size 24 h later (T1-T3; r = − 0.45). Sham DN demonstrated a large effect size to increase mechanical pain sensitivity immediately after the intervention (T1-T2; r = − 0.67) and this large effect size persisted 24 h later (T1-T3; r = − 0.64; see Table 2).

### Neurophysiological and psychophysical relationship

**Fig. 2:**
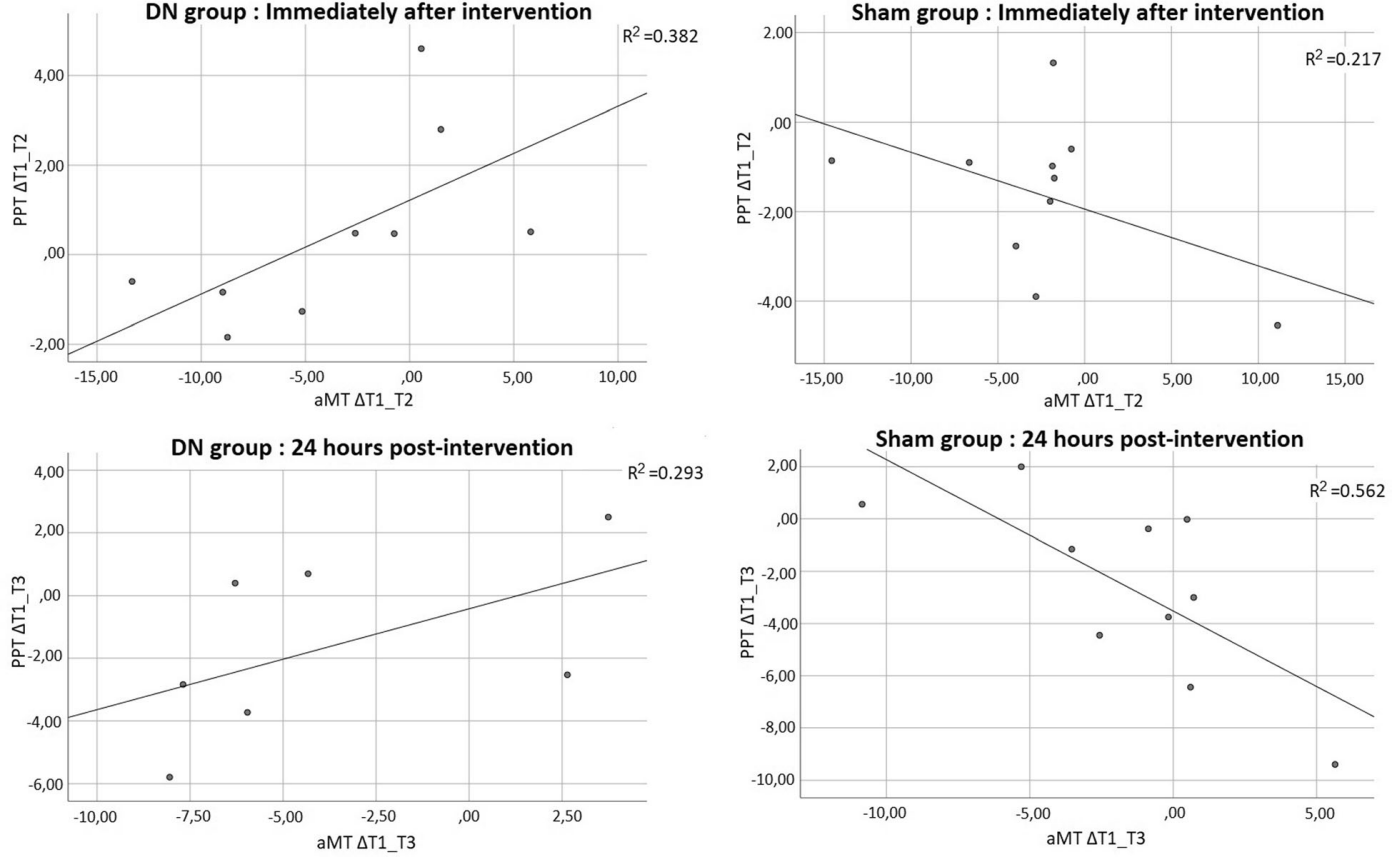
Relationship between neurophysiological and psychophysical outcomes Active motor threshold (aMT) results are expressed as a percentage of the maximum stimulator output (%MSO); Pressure pain threshold (PPT) results are expressed in kg/cm^2^; ΔT1_T2: Delta score between baseline and immediately post-intervention; ΔT1_T3: Delta score between baseline and 24 h post-intervention

No significant correlation was observed in either group between corticospinal excitability and PPT at baseline (T1), immediately post-intervention (T2) and 24 h post-intervention (T3). However, in the DN group, Spearman’s correlation analysis revealed the presence of a significant and positive correlation between delta scores, reflecting corticospinal excitability changes and mechanical pain sensitivity changes between T1 and T2 (r = 0.77, *p* = 0.02) and between T1 and T3 (r = 0.75, *p* = 0.05). This correlation suggests that an increase in corticospinal excitability (reduced aMT) following the DN intervention was associated with an increase in mechanical pain sensitivity (reduced PPT). A significant relationship between corticospinal excitability changes and mechanical pain sensitivity changes was also noted in the sham group between T1 andT3; interestingly, the relationship between these changes was negative (r = − 0.70, *p* = 0.03). This correlation indicating that participants in the sham group with the greatest increase in corticospinal excitability (reduced aMT) were those with the smallest increase or even a decrease in mechanical pain sensitivity. No significant relationship was noted in the sham group for the changes observed between T1 and T2 (r = − 0.08, *p* = 0.83; see Fig. 2).

## Discussion

This study is, to our knowledge, the first to assess the feasibility of a research protocol to assess corticospinal excitability of the infraspinatus muscle following DN and sham DN in a sample of participants with chronic non-traumatic shoulder pain. This research is also the first to report results about the plausible effects of DN on corticospinal excitability and on the relationship between these neurophysiological effects and psychophysical effects.

### Feasibility outcomes

In this study, an exclusion rate of 43% may seem relatively high but is similar to other studies investigating DN [39]. This high exclusion rate *before* the first appointment reflects our conscious choice to only include brief and general information about the clinical portrait of the targeted population on our advertising posters. These posters caught the interest of many potential participants, but ultimately led to a high proportion of ineligible people due to our strict criteria. The presence of an infraspinatus MTrP in 95% of the individuals evaluated in this study is higher than the prevalence rate of 77% reported for the same population by Bron et al. (2013) [6]. This difference might be explained by our strict exclusion criteria since Bron et al. did not exclude confounding pathologies that may lead to chronic shoulder pain but which are not associated with the presence of MTrP (e.g. radiculopathies).

Among the eligible participants contacted, only 4% declined to participate due to an unwillingness to undergo the intervention, 7% because they had apprehension regarding TMS and 7% because their schedule did not allow them to attend the 2 sessions within a 24-h period. A 7-day post-intervention follow-up could potentially reduce this refusal rate, but could also increase loss to follow-up [40]. We were able to recruit 21 participants within the time period initially set at 6 months.

Nineteen of the 21 participants enrolled in this study completed the 24-h post-intervention follow-up. One participant withdrew from the study after experiencing a mild headache following T2. Similar discontinuation rates (4.8%) due to TMS adverse effects have been observed in other studies [41]. An overall loss to follow-up rate less than 10% was considered acceptable [40].

We were only unable to produce discernible MEP amplitudes in one participant in this study. Although issues related to completeness are rarely described in TMS studies (including those examining shoulder MEP measures [26, 36, 42, 43]), the inability to produce discernible MEP is somewhat frequent in human studies. This is a phenomenon that can be explained by subject-specific characteristics and M1 gyral folding pattern variations which, in some individuals, can contribute to a difficulty in producing discernible MEP [44]. An overall completeness rate of 81% was reached, with 70% completeness in the DN group and 91% in the sham group: Without questioning feasibility, this may guided sample size of future studies.

Following DN, mild adverse effects (e.g. bruising or post-needling soreness) lasting up to 36 h are commonly reported and significant adverse effects are rather rare [45].

Specifically, techniques to elicit a local twitch response (LTR) are suspected to be responsible for some of the pain reported post treatment [46]. No adverse effects related to DN were reported in this study despite all participants in the DN group experiencing a single LTR following the procedure described above. Only one participant reported an increase in pain, which was persistent 24 h after the intervention. However, this was considered a TMS-related adverse effect, since this initial increase in pain appeared while doing the isometric external rotation movement during the TMS procedure at baseline (before intervention). Indeed, the position of the participants and the isometric muscle contraction (sustained contraction of a muscle with MTrP and the inevitable co-contraction of other muscles) used in this study to obtain discernable MEPs can put some stress on the tissues of the injured shoulder [47, 48]. In this context, nearly 20% of our participants, including the participant with pain lasting up to 24 h, reported difficulty maintaining the muscle contraction due to discomfort. Having these participants take short, repeated breaks during the procedure made it possible for the researchers to complete the measurements. These participants were not systematically those who reported higher pain intensities (at rest or during activity) at baseline. We suggest incorporating buffer time periods into future studies to enable participants to rest and avoid symptom exacerbations. TMS safety is supported by published meta-analyses and guidelines [25, 41, 49]. Adverse effects related to TMS are generally transient with a largely predictable evolution in resolution [25, 41, 49]. Only 1 participant reported a mild headache without any other concomitant symptom. This resulted in the inability to take and record measurements (withdrew consent). Mild to moderate headache is the most commonly reported TMS adverse effect [25, 41, 49].

### Neurophysiological outcomes

In this present study, DN increased corticospinal excitability (reduced aMT) 24 h post- intervention while sham needling increased corticospinal excitability immediately after the intervention. Previous TMS studies have generally reported variability in the effect of clinical pain on corticospinal excitability and was dependent upon painful conditions [50, 51]. Chronic shoulder pain seems to involve decreased corticospinal excitability of the infraspinatus [26, 34]. Therefore, the results of this study suggest that DN and sham needling could possibly modify central motor alterations associated with chronic shoulder pain.

Our results did not demonstrate a significant difference between the experimental group and the control group. It should, however, be specified that even if the sham needling intervention was not deep enough to reach the MTrP, inserting a needle into the skin’s surface induces effects. Sham needling is known to have an effect on corticospinal excitability [52–56], which would be attributable to a bottom-up effect on the CNS by stimulating sensory-discriminative pathways [54, 57]. Since the effect size of the dry needle puncture intervention is small when compared to a control group receiving a sham [54, 57, 58], the absence of a significant difference between the groups may indicate a lack of power to measure the effect of the intervention rather than evidence that these two modalities are equivalent. In fact, an a posteriori analysis showed 1-β = 0.063 for T1-T2 and 1-β = 0.142 for T1-T3 for this outcome. Moreover, the results of this study suggest that DN and sham needling could increase corticospinal excitability of the infraspinatus with a different pattern over time. As shown by the effect size (see Table 2), a DN effect on corticospinal excitability appears to increase over time while a sham needling effect appears to decrease. It is possible that similar, nonspecific, somesthetic stimuli between DN and sham needling may explain the initial effect observed in both groups [57, 59],while the absence of a therapeutic dose [59] and a gradual decrease in the placebo effect [60] may explain the subsequent decrease in the effect observed in the sham group. For this reason, future DN studies should consider incorporating more than one short-term follow-up as well as incorporating long-term follow-up.

### Psychophysical outcomes

In the present study, both groups showed decreased PPT (increased mechanical pain sensitivity) immediately after the intervention and 24 h post-intervention compared to baseline; however, these differences were only significant in the sham group. Statistically, significant between-group differences were only observed immediately after the intervention, possibly demonstrating increased sensitivity in the DN group over time (as shown by T1-T3 median [IQR] and effect size; see Table 2). It is important to note that many studies reported that DN and sham needling increase PPT (decreased mechanical pain sensitivity) in the short term [13, 61], although some do not detect a significant effect [62]. In this study, in order to obtain free and informed consent, each participant was verbally informed before needle insertion that the technique could be sensitive and cause soreness lasting up to 48 h. Expectations are recognized as an important factor influencing pain modulation [63], particularly during puncture therapy [57]. Thus, these results may partially be explained by a nocebo effect generated by negative expectations associated with these instructions given before the procedure [57, 63]. It is possible that the therapeutic effects of DN on a biochemical MTrP environment [64–66] had partly counterbalanced the nocebo effect of negative expectations in the DN group, which explains the difference observed the between groups. As these effects would be short term [64], this hypothesis would be consistent with the possible increased sensitivity observed in the DN group at T3. Given the importance of expectations associated with puncture treatment previously mentioned, we consider that future DN studies should quantify participants’ expectations before the intervention.

### Neurophysiological and psychophysical relationship

Previous studies in healthy subjects [67] and in patients with chronic pain [68, 69] reported a relationship between neurophysiological and psychophysical aspects. Although no relationship between corticospinal excitability and PPT measurements was detected in the present study, delta scores of these two outcomes indicate in the DN group that a greater increase in corticospinal excitability is associated with a greater increase in mechanical pain sensitivity, and conversely in the sham group, that a larger increase in corticospinal excitability may be associated with a smaller increase in mechanical pain sensitivity.

This different correlation between corticospinal excitability and mechanical pain sensitivity is possibly a first step in dissociating the predominant and non-exclusive bottom-up effects of DN compared to sham needling. Puncture, even superficial puncture, is known to activate low threshold mechanosensitive C-fibers related to gentle touch which are represented by a specific pathway that exerts a complex affective-emotional reaction [19, 57] which, in turn, is known to modulate analgesic top-down mechanisms [63, 70]. On the other hand, puncture is also associated with an activation of the pain matrix (sensorimotor cortical network, including the insula, thalamus, and anterior cingulate cortex, as well as both the primary and secondary somatosensory cortices) [57]. In the present study, the needle insertion depth (not the insertion location) differentiated the sham needling from the DN intervention. From a mechanistic perspective, we can consider that these two modalities thus activated different sensory receptors associated with different layers of tissues stimulated. Consequently, we can ultimately hypothesize that DN and sham needling activated these previously described pathways to a different degree. Sham needling may predominantly rely on tactile C-fibers and the motivo-affective component modulation of pain, explaining why increased corticospinal excitability was negatively correlated to mechanical pain sensitivity. On the other hand, DN may predominantly rely on the activation of the pain matrix resulting in increased motor cortex excitability as a mechanism aimed at reducing thalamic overactivity and thus pain [68, 71]. This may explain why increased corticospinal excitability was positively correlated to mechanical pain sensitivity in this case. To verify this hypothesis, it would be relevant that future DN studies quantify participants’ perceived pain and unpleasantness during the intervention.

### Limitations

This study initially intended to assess feasibility outcomes; therefore, corticospinal excitability and mechanical pain sensitivity results must be interpreted with caution. Due to the low statistical power, this study presents a high risk of type II errors where the null hypothesis is not rejected despite being false. Therefore, a larger sample would be required to properly investigate the difference between real and sham DN, and their respective effects. Moreover, this study did not measure patient functional abilities or the long-term effects of DN. It should also be noted that this study was retrospectively registered. However, no significant changes were made during the study.

Only the aMT was evaluated as a measure of corticospinal excitability. This measurement is a specific but not sensitive way to quantify corticospinal excitability [30]. It also assesses the corticospinal pathway without differentiating where, along this descending pathway, changes occur. It is suggested that MTrP implies a locally biochemical imbalance and hyperactivity at the neuromuscular junction (NMJ) [5, 72]. Most methods used to assess and dissociate spinal and peripheral components from the central components of the corticospinal pathway require stimulation of the peripheral nerve [73, 74]. Although techniques to stimulate the suprascapular nerve (which innervates the infraspinatus) have been described [75, 76], several limitations remain [76, 77], especially in a context where insertion of a needle is the investigated intervention and thus cannot be an option for taking measurements. DN seems to favorably modulate the biochemical environment of MTrP [65, 78] and decrease NMJ hyperactivity [23]. Therefore, it would be relevant to take other measurements, including TMS corticospinal excitability measurements, dissociating spinal and/or peripheral components of the nervous system (e.g. compound motor action potential, H reflex) in future studies evaluating the effects of DN applied in muscles that allow these kinds of measurements (e.g. calf muscle). Such studies would help to determine whether the changes observed are exclusive or combined between these different components of the nervous system.

## Conclusion

The present study demonstrates that measuring the neurophysiological and psychophysical effects of DN is feasible. It provides recommendations and guidelines for future studies as well as preliminary evidence on these neurophysiological and psychophysical effects and their relationships. DN and sham DN applied in MTrP infraspinatus seem to both increase corticospinal excitability. The hypothesis that this effect following both interventions could be different in intensity and over time cannot be undoubtedly rebutted (given the lack of power for this outcome). However, it should be noted that we did not achieve statistical significance when comparing the groups. Nevertheless, the observed relationship between changes in corticospinal excitability and sensitivity to mechanical pain suggests a difference in the effects of these two techniques. Future studies investigating these effects and their relationships will be needed and should consider participants’ expectations, long-term follow-up, functional measures, and may also extend to muscles which proximal nerve conduction studies are valid and reliable with surface electrodes.

## Data Availability

The datasets used and analyzed during the current study are available from the corresponding author upon reasonable request.

## Abbreviations

aMT: Active motor threshold
CNS: Central nervous system
DN: Dry needling
EMG: Electromyography
ES: Effect size
IQR: Interquartile range
LTR: Local twitch response
Mdn: Median
MEP: Motor evoked potential
MSO: Maximum stimulator output
MTrP: Myofascial trigger point
NMJ: Neuromuscular junction
PPT: Pressure pain threshold
TMS: Transcranial magnetic stimulation
VAS: Visual analog scale
